# Human papillomavirus DNA detection in plasma and cervical samples of women with a recent history of low grade or precancerous cervical dysplasia

**DOI:** 10.1371/journal.pone.0188592

**Published:** 2017-11-28

**Authors:** Clementina Elvezia Cocuzza, Marianna Martinelli, Federica Sina, Andrea Piana, Giovanni Sotgiu, Tiziana Dell’Anna, Rosario Musumeci

**Affiliations:** 1 Department of Medicine and Surgery (School of Medicine), University of Milano-Bicocca, Monza, Italy; 2 Obstetrics and Gynaecology Department, San Gerardo Hospital, ASST Monza, Monza, Italy; 3 Department of Biomedical Sciences, University of Sassari, Sassari, Italy; Universidade Estadual de Maringa, BRAZIL

## Abstract

Circulating HPV DNA has been previously described in women with advanced stages of cervical cancer and has been suggested to be a prognostic marker of disease recurrences and metastases. Only a few studies have reported the presence of HPV DNA in bloodstream of patients with low grade or precancerous cervical lesions. This study aimed to define if HPV DNA could be detected in plasma samples of 120 women referred for a recent history of cervical dysplasia who presented with lesions ranging from High Squamous Intraepithelial Lesion (H-SIL) to regressed normal cytology. HPV DNA detection was carried out in both plasma and cervical samples using type-specific real-time quantitative PCR assays identifying oncogenic HPV 16, 18, 31, 33, 45, 51 and 52.

Overall, 34.2% (41/120) of plasma samples were shown to be positive for HPV DNA detection; HPV 45 (46.3%), HPV-51 (29.6%), and HPV 16 (18.5%) were the most frequently identified genotypes. The rate of HPV detection in paired cervical and plasma samples increased with advancing disease stage, ranging from 15.4% in women with regressed lesions to 38.9% in women with HSIL; HPV 16 resulted the most common genotype identified in women found to be HPV DNA positive in both cervical and plasma samples. Moreover, HPV 16 showed the highest median viral load value in both cervical and plasma samples, with 48,313 copies/10^4^ cells and 1,099 copies/ml, respectively.

Results obtained in this study confirm that HPV DNA can be detected and quantified in plasma samples of women with asymptomatic cervical infection. Further knowledge on HPV dissemination through the blood stream of women with cervical lesions would be very important in better understanding the natural history of HPV infection as well as its potential role in other distant tumors.

## Introduction

Cervical Cancer (CC) is one of the most incident cancers in women worldwide, especially in developing countries, with an estimated incidence of 528,000 in 2012 [1]. Its mortality rate follows that associated with breast cancer among women aged less than 45 years in Europe [2].

Infection with high-risk human papillomavirus (hr-HPV) genotypes has been well recognized to be causally associated with almost all CC types. The integration of hr-HPV DNA into the cellular genome and the overexpression of HPV E6 and E7 oncogenes are deemed responsible for different cellular pathways alterations [3]. As these changes are critical for the cellular transformation and proliferation, detection of hr-HPV DNA in cervical samples has been demonstrated to be a useful viral marker in CC screening [4]. Furthermore, hr-HPV viral load has been associated with a cervical lesion progression or regression [5].

Infection with hr-HPV has also been associated with the development of oropharyngeal cancer, as well as, more recently, with the occurrence of other extra-genital tumors, such as breast, lung, bladder, and colon cancers [6–9]. How the virus disseminates to anatomical sites distant from the genital mucosal surfaces, where viral replication occurs, is still controversial, although the haematogenous spread has been proposed. Previous studies have reported the presence of HPV DNA in peripheral blood mononuclear cell (PBMCs), sera and plasma samples [10–16]. Most of these studies have been conducted recruiting women with diagnosed CC caused by HPV 16 and /or HPV18; authors underscored the association of circulating viral DNA detection with the lysis of cancer cells or with micrometastases shed by the cervical tumour [13–17]. Moreover, some investigators have described the potential role of circulating HPV DNA not only as marker of CC but also of lung and breast cancers, suggesting that bloodstream could be the vehicle used by the virus to reach anatomical sites far from the cervical mucosa [9]. Only few studies have investigated the presence of HPV DNA in bloodstream of “non-cancer” patients with asymptomatic urogenital HPV infections and/or low grade precancerous cervical lesions, usually consider these subjects as a “control group” [13–17].

In order to better understand the association between bloodstream circulating HPV-DNA and cervical infection in women presenting with a recent history of cervical dysplasia, this study aimed to assess the presence (qualitative and quantitative) and agreement between both plasma and cervical samples of seven of the most prevalent hr-HPV types in our geographical area.

## Material and methods

### Ethics statement

The study protocol was approved by the Ethics Committee of San Gerardo Hospital, Monza, Italy (Protocol: 08/UNIMIB-HPA/HPV1; n. 1191). All subjects provided written informed consent to participate in the study.

### Study design and sample collection

A group of women with a recent diagnosis of cervical dysplasia attending the Gynaecology Out-Patients Clinic of San Gerardo Hospital, Monza, Italy were enrolled in the study. Following routine gynaecological examination and repeated Pap smear examination, additional blood and cervical samples were collected from each patient for hr-HPV detection.

Immunocompromised patients, women with autoimmune diseases or any diseases involving the immune system, with HIV infection, previously vaccinated against HPV, with a presumed or confirmed pregnancy, with a diagnosis of any malignancies, undergoing or having finished a course of chemotherapy during the six months preceding the study were excluded from the study. Women with a negative Pap test were selected as a control group.

Paired cervical liquid cytology and blood samples were collected on the same day from all women visiting the Gynaecology and Obstetrics Department of the San Gerardo Hospital, Monza, Italy.

Cytological diagnosis of cervical lesions was performed at the hospital, and findings were assessed according to the 2001 Bethesda System for cervical cytological reporting [18].

Plasma and cervical samples were tested for HPV DNA at the Clinical Microbiology Laboratory of the Department of Medicine and Surgery, University of Milano-Bicocca, Italy.

### Study materials

#### Cervical samples

Cervical cytology samples were collected using the Abbott cervi-collect specimen collection kit (Abbott) and transported in ThinPrep® PreservCyt® Solution (HOLOGIC™). 15 ml of cervical samples were centrifuged at 2000 rpm for 15 minutes at 4°C for cervical cells concentration. Cell pellet was suspended in 2.5 ml of PBS and 5 aliquots of 500 μl for each samples were made. One aliquot was used for DNA extraction.

#### Blood samples

Blood samples were obtained by venepuncture from patients using lithium-heparin anti-coagulant and transported to the laboratory at 4°C. In the laboratory, 20 ml of blood were slow pipetted to 20 ml of Lympholyte®-H, sterile liquid (EuroClone) and centrifuged at 400 xg for 40 minutes without brake to obtain blood stratification. Approximately 6 mL of plasma were transferred in a 15 mL centrifuge tube and stored at -80°C. One ml of each sample was used for subsequent DNA extraction.

### DNA extraction

DNA extraction from a 500 μl cell pellet aliquot of cervical sample was carried out using CLART® HPV2 Extraction-Purification and Amplification kit (Genomica) following manufacturer protocol.

DNA extraction from 1 mL of plasma sample was performed using NucliSENS® easyMag® (bioMérieux), a second-generation system for automated isolation of nucleic acid from clinical samples. All samples were processed using “specific” protocol, characterized by a higher final elution temperature (about 70°C) and the use of silica beads diluted 1:2, in accordance to the manufacturer’s instructions. The nucleic acids extraction system is fully automated, except for adding the initial sample of plasma (1 ml) and the silica beads (125 μl) 10 minutes after sample lysis, reducing potential risks of sample cross-contamination. The nucleic acids extracts were eluted in 70 μl of NucliSENS® easyMag® elution buffer (bioMérieux).

### HPV detection and quantification

HPV DNA amplification was carried out using previously described “in-house” Real-Time quantitative TaqMan PCR assays [19–22]. The hr-HPV genotype-specific assays allow detection and quantification of HPV 16, 18, 31, 33, 45, 51 and 52 DNA, these being among the hr-types most frequently associated with cervical cancer development in our geographical area. C-C chemokine receptor type 5 (CCR5) gene amplification and quantification was also carried out on cervical samples’ nucleic acid extracts in order to determine sample’s cellularity and normalize HPV viral loads [19]. Amplification was performed using ABI Prism device (7900 SDS; Applied Biosystems). The thermal cycling conditions, optimized to obtain the best amplification kinetics under the same temperatures and composition of reaction mixture, consisted of the following thermal profile: 2 minutes at 50°C, 10 minutes at 95°C and 40 cycles of 15 seconds at 95°C and 1 minute at 60°C. Genotype-specific hr-HPV viral loads are expressed as copy numbers for ml in plasma samples and as copy numbers/10^4^ cells in cervical samples.

All HPV Real-Time quantitative TaqMan PCR assays were previously validated by participation to WHO HPV Laboratory Proficiency Lab-Net 2014.

### Statistical analysis

Data were collected on standardized e-forms and analysed using Stata 9.0 (StataCorp, Stata Statistical Software Release 9, College Station, TX, USA 2005). Qualitative and quantitative variables were summarized with absolute (relative) frequencies and medians (interquartile ranges, IQR), respectively. A two-tailed p-value less than 0.05 was considered statistically significant.

## Results

### HPV detection in cervical samples

A group of 120 women (median, IQR: 35.5, 27–43.25 years) was enrolled. Twenty women were recruited as control group.

Normal cervical cytology was detected in 21.7% (26/120) of recruited women with a documented abnormal Pap test in the preceding 6 months (“Regressed”). Abnormal Pap smears at enrolment were detected in 78.3% of the study participants: 25.5% (24/94), 55.3% (52/94), and 19.2% (18/94) showed atypical squamous cells of undetermined significance (ASCUS), low grade squamous intraepithelial lesion (LSIL), and high-grade squamous intraepithelial lesion (HSIL), respectively.

**Fig 1:**
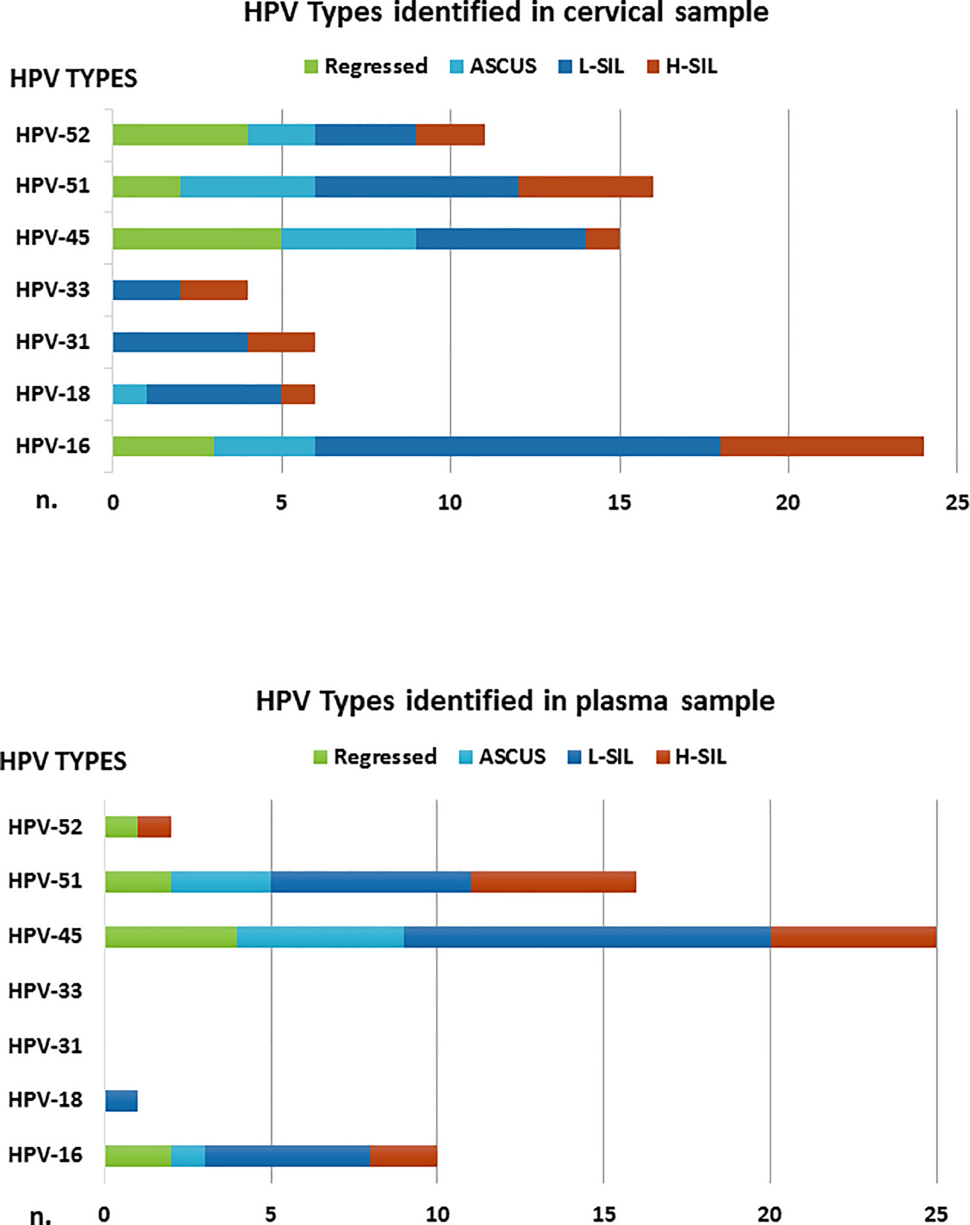
HPV genotypes distribution in cervical and plasma samples. Correlation of HPV types identified in cervical and plasma samples with cervical cytology.

Overall, 44.2% (53/120) of selected women were found to be positive for the detection of one or more of the 7 hr-HPV types investigated. 37.7% (20/53) of HPV DNA positive women showed infection with multiple HPV-types for a total of 82 HPV cervical infections. Data regarding the genotypes identified in cervical samples are reported in Fig 1. The most prevalent genotypes were: HPV 16 (29%; 24/82), HPV-51 (19%; 16/82), and HPV 45 (18%; 15/82). HPV 16 showed the highest viral load with a median value of 48,313 copies/10^4^ cells (IQR: 1,634–416,066), followed by HPV 51 (30,609 copies/10^4^ cells; IQR: 2,190–67,068) and HPV 45 (3,500 copies/10^4^ cells; IQR: 469–141,756).

HPV DNA was detected in 42.3% (11/26) of women with regressed lesions, 33.3% (8/24) with ASCUS, 42.3% (22/52) with LSIL, and 66.7% (12/18) with HSIL, respectively (Table 1). No positivity for HPV DNA was detected in the control group.

**Table 1:** Correlation of HPV positivity identified in cervical and plasma samples and cervical cytology.

Cervical Cytology	Patients		Median Age (IQR*)	HPV positive– Plasma samples		HPV positive - Cervical samples	
	N.	%	years	N.	%	N.	%
**Normal** Control Group	20	14.3	31.5 (25–37)	0	0	0	0
**Normal** Regressed Lesion	26	18.5	39.5 (30.25–48)	8	30.8	11	42.3
**ASCUS**	24	17.1	34 (28.5–45.25)	8	33.3	8	33.3
**L-SIL**	52	37.1	31 (24–39.5)	16	30.8	22	42.3
**H-SIL**	18	12.9	31 (27.5–43.5)	9	50.0	12	66.7
**Total**	**140**	**100**	**35.5 (27–43.25)**	**41**	**29.3**	**53**	**37.9**

*IQR: interquartile ranges

### HPV detection in plasma samples

Overall, 34.2% (41/120) of women were found to be positive in plasma samples for 5 out of the 7 hr-HPV types investigated (*i*.*e*., HPV 16, 18, 45, 51 and 52); nine of these women (22%; 9/41) showed the presence of multiple HPV-types for a total of 54 HPV-positivities. Genotypes detected in plasma samples are described in the Fig 1. The most prevalent genotypes were: HPV 45 (46.3%; 25/54), HPV-51 (29.6%; 16/54), and HPV 16 (18.5%; 10/54). HPV 16 showed the highest viral load also in plasma samples with a median value of 1,099 copies/ml (IQR: 616–3,036), followed by HPV 45 (median value: 418 copies/ml; IQR: 286–1,066) and HPV 51 (median value: 286 copies/ml; IQR: 154–5,281).

HPV DNA was found in 30.8% (8/26) of women with regressed lesions, 33.3% (8/24) with ASCUS, 30.8% (16/52) with LSIL, and 50.0% (9/18) with HSIL, respectively (Table 1).

All samples from women in the control group were found to be HPV DNA negative.

### Agreement between HPV detection in cervical and plasma samples

Twenty-five (25/120; 20.8%) women were found to be HPV-DNA positive both in cervical and plasma samples; among them 44% (11/25) were shown to have the same HPV genotype in both samples (Table 2). HPV-16 was the genotype most commonly detected in women positive in both cervical and plasma samples (5/25; 20%).

**Table 2:** Profile of women (n. 25) that showed hr-HPV DNA presence in both cervical and plasma samples.

ID Patient	Age (years)	Cytology	HPV plasma	HPV cervix
**31**	45	Regressed	45	45
**77**	54	Regressed	51	16
**78**	45	Regressed	45	16,52
**80**	32	Regressed	16,45	52
**5**	32	ASCUS	45	51
**11**	34	ASCUS	51	51
**169**	40	ASCUS	45	16,18,45,51,52
**195**	65	ASCUS	45	16
**13**	26	L-SIL	51	51
**23**	36	L-SIL	45,51	16
**41**	47	L-SIL	16	16,51
**60**	50	L-SIL	16	16
**66**	36	L-SIL	45	16,18
**77 T6**	54	L-SIL	45	16
**85 T6**	55	L-SIL	45	16
**96**	42	L-SIL	16,18,45,51	16,18
**97**	26	L-SIL	45,51	16,33,45,51,52
**152**	26	L-SIL	16,45,51	16,45
**35**	49	H-SIL	45	16
**41 T6**	47	H-SIL	51	52
**94**	35	H-SIL	16	16
**120**	55	H-SIL	45,51,52	16
**158 T6**	52	H-SIL	51	16,31,51
**176**	65	H-SIL	45	16
**178**	58	H-SIL	51	33

**Fig 2:**
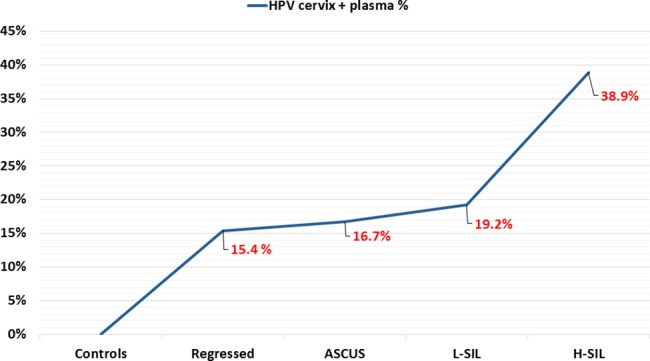
Correlation of HPV detection in plasma with the cervical cytology. Graph shows the percentage of women resulted positive for hr-HPV DNA detection in both cervix and blood related to cervical cytology.

Concomitant detection of the 7 hr-HPV types investigated in both cervical and plasma samples increased with the severity of the disease, ranging from 15.4% (4/26) in women with normal cytology/regressed lesions to 38.9% (7/18) in women with HSIL (Fig 2).

Thirteen percent of women (16/120) showed HPV positivity only in plasma samples: the most frequently detected genotypes were HPV 45 (9/16, 56.3%), HPV 51 (5/16, 31.3%), and HPV 16 (4/16, 25.0%). Twenty-eight women (28/120; 23.3%) were HPV DNA positive only in cervical samples: the most frequently detected genotypes were HPV 45 (11/28, 39.3%) HPV 51 (10/28, 35.7%), and HPV 16 (6/28, 21.4%). The remaining 51 patients resulted negative for the investigated 7 hr-HPV in both sample types.

## Discussion

HPV are well-known epitheliotropic viruses requiring differentiating squamous epithelium for their life cycle. The oncogenic potential of hr-HPVs has been associated with the occurrence of cervical, other ano-genital as well as head and neck cancers [23, 24]. More recently, hr-HPV types have also been found in other tumors, such as breast [8, 25], lung [8, 9], colon [24], and oesophageal cancers [26]. In addition, some reports demonstrated the presence of HPV DNA in subjects with no recognized HPV-related disease or cancer, with viral nucleic acids being detected in sperm cells [27,28], placental tissue [29,30], or PBMCs [10, 11]. These findings seem to indicate that HPV can spread to extra-genital sites through the bloodstream [27, 31].

Limited data are available on the presence of HPV DNA circulating in the bloodstream. Previous reports have described only a limited number of oncogenic HPV types, mostly HPV-16 and -18, in blood samples of women with CC. Moreover different studies investigating the presence of HPV DNA in plasma of women with CC have demonstrated very different PCR detection rates ranging from 6.9% (11/175) HPV 16/18 positivity, as compared to 1.7% (1/60) positivity in controls [16]; 12% (6/50) HPV 16/18 positivity (0% in 20 controls) [32]; 13.7% (8/58) HPV 16/58 positivity (0% in 30 controls; 0% in 10 CIN) [13]; 50% (57/114) HPV 16/18 positivity [17] up to 65% (11/17) HPV 16/18/31/33/58 positivity [12]. Varying reported plasma HPV positivity rates in women with cervical cancer could be accounted for by the number of hr-HPV types analysed as well as on differences in technical aspects, such as: plasma starting volumes (ranging from 200ul to 1ml), viral nucleic acid extraction methods and analytical sensitivity of the molecular assays [12, 33]. A more recent study, conducted by Jaennot et al. [34], using the highly sensitive droplet PCR method, showed a high positivity rate (87.1%, 61/70) in serum samples of patients with HPV-associated cervical, anal, and oropharyngeal cancers. This study also described similar detection rates in both plasma and serum samples, as well as demonstrating improved detection in samples stored at -80°C as compared to -20°C [34].

These studies, together with other reports [9, 35, 36], suggest that the detection of HPV DNA in blood samples could be a useful marker of severe HPV-related cancers, metastatic disease, or disease recurrences.

Only few studies have previously reported HPV detection in blood samples of women and men with non-cancer related HPV urogenital infections. Pao et al. reported HPV DNA detection in PBMCs of 52% (13/25) of asymptomatic women with urogenital infections [11]; Foresta et al reported 25% (4/16) positivity in blood of male subjects with HPV-16 semen infection [27] and Chen et al in 8.3% (15/180) of healthy Australian male blood donors [10]. These reports suggest that HPV dissemination via the bloodstream may occur even during transient asymptomatic infections and may account for the oncogenic role of hr HPV in extra-genital tumours.

On this basis, we investigated the presence of HPV DNA in blood samples of 120 asymptomatic women presenting with a recent history of cervical dysplasia. Detection of HPV DNA was performed by the use of previously described highly sensitive hr-HPV type-specific quantitative real-time PCR assays [19–22]. Overall, 34.2% (41/120) of plasma samples were shown to be positive for HPV 16 and/or 18, 45, 51 and 52. This relatively high positivity rate is in contrast with results obtained in previous studies investigated HPV positivity in blood of women with cervical cancer, where patients with pre-cancerous lesions were mainly included as “controls” [13–17, 32]. Moreover most previous studies focused their research only on detection of HPV 16 and HPV 18, whilst in this study we extended the investigation to include seven of the most prevalent hr-HPV types in our geographical area.

Concomitant HPV detection in both cervical and plasma samples was found to increase with the severity of the cervical dysplasia, ranging from 15.4% to 38.9%. Forty percent of women with HPV infection both in cervix and in plasma showed the same genotype, with HPV-16 (20%) being the most commonly identified HPV type. This is in keeping with results of previous studies on women with CC, also reporting HPV-16 as the more prevalent genotype both in blood and cervical samples [13, 16], although fewer HPV genotypes were being investigated. In our study however the hr-HPVs detected in plasma samples did not always correlate with the HPV types detected in the cervix (56%; 14/25). Although most previous studies investigating HPV types in paired cervical and blood samples showed 100% agreement[13, 14], Dong et al. also detected different HPV types in plasma and genital tract samples in 35.7% (5/14) of women with CC [16]. Moreover in our study, most hr-HPV cervical positivities were associated with low grade cervical dysplasia, which is more likely to be associated with transient infections. The discrepancy between plasma and cervical HPV positivity observed in our study could therefore be explained by the high frequency of transient HPV cervical infections, often associated with multiple types, as well as with potential hr-HPV infection in sites other than the genital mucosa [10, 11, 34]. These infections may independently result in viral persistence and/or replication in the bloodstream [31, 37]. Moreover the under reported discrepancies between cervical and blood HPV detection may also in part be explained by the relatively few hr-HPV types investigated in previously studies [13, 14, 16].

Our study also showed 16 women to be HPV DNA positive only in plasma samples, in the absence of cervical infection. Again, this could result from a cleared cervical infection and/or HPV presence in other non-genital mucosal sites associated with viral persistence and/or replication in the bloodstream [31, 34, 37]. It has been previously suggested that other cells, such as PBMC, may in fact be permissive to papilloma virus replication [37]. Further studies investigating for the presence of whole virus in the blood cellular compartment or for evidence of viral replication through the expression of oncogenic transcripts, will help to clarify if these cells can just act as carriers for viral spread or if they can also be permissive for HPV replication and persistence. This would have important implications in contributing to both the nonsexual spread and to tumor development in anatomical tissues far from the mucocutaneous sites of primary viral replication.

Quantification of HPV viral load in cervical samples could represent a potential marker to discriminate persisting from regressing cervical infections. Data supporting this correlation and HPV viral load prognostic significance have been previously reported [5, 38].

Regarding the role of HPV viral load in blood, few studies have reported a possible correlation between HPV viral load in plasma and tumor clinical stage, suggesting that it could represent a possible marker for advanced cervical disease and/or a genetic marker associated with CC metastasis/recurrences [16, 17, 32]. In this study, HPV type-specific viral load were evaluated only for the most frequently identified genotypes. HPV 16 showed the highest median viral load both in cervical and plasma samples. The HPV 16 median viral load identified in blood (1,099 copies/ml) is higher than that reported in previous studies. For example, Ho et al. [15] reported a median value of 586 copies/ml, Gnanamony et al. [17] a median value of 253 copies/ml and Dong et al. [16] a median value of 183 copies/ml. No statistical significance was observed between viral load and grade of cervical lesion (data not shown), probably due to the low number of data used for the analysis. Further future longitudinal studies with a larger number of patients could clarify the clinical significance of HPV detection and quantification in blood samples.

## Conclusions

This study provides further evidence that HPV DNA can be detected and quantified in plasma samples of women with a recent history of low grade cervical dysplasia and/or cleared genital HPV infection. This is contrast with some previous reports correlating circulating HPV DNA with cervical carcinoma or other HPV-related cancers. The identification of hr-HPV DNA in plasma samples of women with apparently cleared cervical infection provides evidence for possible viral persistence and/or replication in anatomical sites other than the genital mucosa. Longitudinal studies are however required to further evaluate the role of HPV DNA detection in the bloodstream of women with asymptomatic infection and/or early stage cervical dysplasia.
